# THE RELATIONSHIP BETWEEN IMMIGRANT STATUS AND UNDIAGNOSED DEMENTIA

**DOI:** 10.1093/geroni/igz038.429

**Published:** 2019-11-08

**Authors:** Yujin Franco, Eun Young Choi

**Affiliations:** 1 University of Southern California, Los Angeles, California, United States; 2 Davis School of Gerontology, University Southern California, Los Angeles, California, United States

## Abstract

In the U.S., the immigrant population is rising, and immigrants are more likely to develop dementia than the U.S.-born population. However, little is known about the rate of undiagnosed dementia among immigrants. This study investigates the relationship between immigrant status and undiagnosed dementia, using 2011 data from the National Health and Aging Trends Study. Data from 7,347 older adults aged 65 years and older (6,531 U.S.-born and 816 immigrants) were included in the analysis. Study participants were divided based on whether they had or had not been diagnosed with dementia, respectively. The results of binary logistic regression showed that being an immigrant was associated with two times higher odds (odds ratio [OR]: 2.00, 95% CI: 1.38-2.92) of undiagnosed dementia compared to US-born participants. Among immigrants, undiagnosed participants had significantly lower levels of depression (t(166)=-2.60, p=.01). Moreover, although marginally significant, the latter were younger (t(166)=-1.90, p=.06) and immigrated at an older age (t(159)=1.87, p=.06) than the diagnosed group. Thus, it is important to tailor dementia education and interventions to the immigrant population, as this may contribute to reducing health disparities in dementia outcomes within the older population.

